# Vertical lid split approach for optic nerve sheath decompression

**DOI:** 10.4103/0301-4738.53057

**Published:** 2009

**Authors:** Venkatesh C Prabhakaran, Dinesh Selva

**Affiliations:** Oculoplastic and Orbital Division, Department of Ophthalmology and Visual Sciences, University of Adelaide and the South Australian Institute of Ophthalmology, Adelaide, Australia

**Keywords:** Optic nerve sheath fenestration, vertical lid split

## Abstract

We describe a vertical lid split orbitotomy approach to perform optic nerve sheath fenestration which was done in a patient with idiopathic intracranial hypertension. A vertical lid split incision was used to enter the superomedial orbit and approach the optic nerve sheath. This approach resulted in a successful nerve sheath fenestration, with improvement in the patient's symptoms. The vertical lid split incision provides access to the optic nerve sheath with minimal morbidity and may be an option for optic nerve sheath decompression.

Since DeWecker's original description in 1872,[1] many different surgical approaches have been reported for optic nerve sheath decompression (ONSD). We report a case wherein the vertical lid split orbitotomy was successfully employed to fenestrate the optic nerve sheath.

## Case Report

A 34-year-old overweight woman presented with headache and decreased vision in the left eye over a four-month period. No other risk factors were present for idiopathic intracranial hypertension (IIH). On examination, best-corrected visual acuity was 20/40 in the left eye and 20/20 in the right eye. Color vision was affected in the left eye (Ishihara plates, left eye: 7/13, right eye 12/13). A left afferent pupillary defect was noted. Bilateral papilledema was present, left worse than the right. Humphrey visual fields revealed an enlarged blindspot in the left eye.

Magnetic resonance venography demonstrated dilated retrobulbar optic nerve sheaths, left more than the right. No mass lesions or dural sinus thrombosis was noted. Lumbar puncture showed an elevated opening pressure. Biochemical investigations of the cerebrospinal fluid (CSF) were normal. A diagnosis of IIH was made and the patient was started on acetozolamide 500 mg twice a day. Ophthalmic review at eight weeks showed mild worsening of visual acuity and color vision in the left eye and early peripheral constriction on the left visual field.

**Figure 1:**
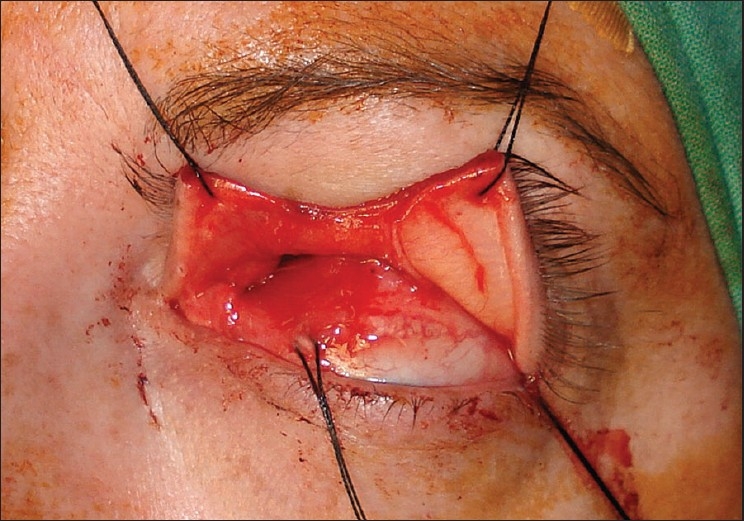
Intraoperative photograph showing vertical split at the junction of the medial and central thirds of the upper lid

**Figure 2:**
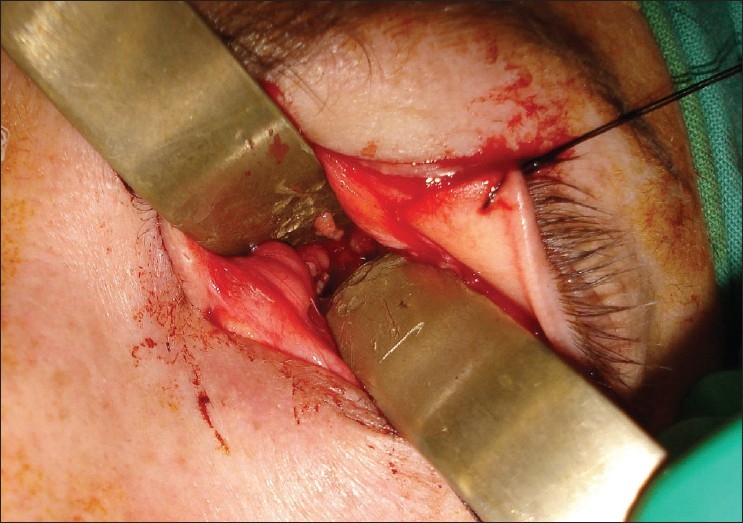
Intraoperative photograph demonstrating approach to the intraconal space via the vertical lid split incision

**Figure 3:**
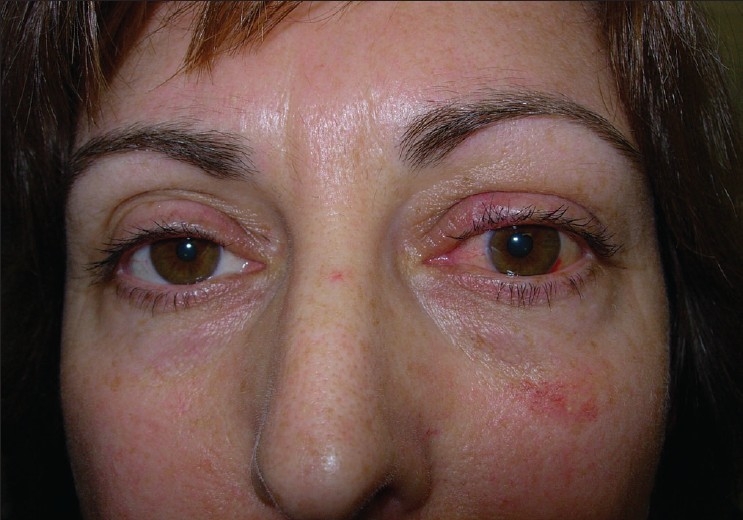
Postoperative photograph showing excellent cosmetic result of the vertical lid split approach (left eye operated)

The patient then underwent a left optic nerve sheath fenestration via a superomedial lid split approach. Under general anesthesia, the left upper lid was infiltrated with local anesthetic with 1:200,000 epinephrine to enhance hemostasis. The medial and superior rectus muscles were secured with 4-0 silk traction sutures to permit globe positioning during surgery. A full-thickness vertical incision was made at the junction of the medial and central thirds of the upper lid. Care was taken to remain exactly perpendicular to the lid margin. Straight iris scissors were used to transect the skin, orbicularis and tarsal plate [Fig. 1]. The incision was extended superiorly through the levator aponeurosis and palpebral conjunctiva up to the superior fornix. The incision was then extended through the conjunctival fornix continuing down to the bulbar conjunctiva lateral to the plica. Blunt dissection was then used to enter the superomedial orbit. The tendon of the superior oblique was identified and retracted medially. The intraconal space was entered between the superior rectus and medial rectus muscles [Fig. 2]. The globe was retracted laterally to enhance exposure. The optic nerve sheath was identified and neurosurgical forceps and scissors were used to open a rectangular window in the dural sheath. Hemostasis was obtained. The bulbar and forniceal conjunctiva were reapproximated with 8-0 polyglactin sutures. The lid incision was repaired in a manner identical to a full-thickness lid margin laceration with 6-0 absorbable polyglactin sutures. The recovery was uncomplicated. Vision in the left eye improved to 20/25; right eye retained 20/20 vision. Visual field changes resolved and papilledema improved markedly in the left eye. The patient remained on medical management and at the last follow-up at 28 months the findings were unchanged. Fig. 3 is the postoperative photograph.

## Discussion

ONSD is performed to decrease the intra-optic nerve sheath CSF pressure in cases of IIH with visual symptoms. The traditional indication for ONSD is progressive visual symptoms (worsening visual acuity or visual field attributable to papilledema) in the absence of significant headache. If headache is prominent then a ventriculo- or lumbo-peritoneal shunt is the procedure of choice.[2]

The commonly used approaches for ONSD are the medial transconjunctival approach and the lateral approach, with or without a bone flap.[3–5] Problems with a medial conjunctival approach include a narrow surgical field, need for disinsertion of the medial rectus and excessive traction on the globe. The lateral approach provides a wider surgical field but is associated with increased tissue dissection (especially when a bone flap is used) and possibility of injury to the ciliary ganglion resulting in pupillary dysfunction. Pelton *et al*. have described a superomedial lid crease approach to the optic nerve sheath.[6] This approach provides a wide surgical field but requires retraction of the medial horn of the levator which may result in a temporary medial ptosis.[6] Also, the surgical field is narrower than that of the lid split approach.

The vertical lid split approach provides good access to the medial intraconal space and is associated with little morbidity. In 1962, Byron Smith originally described this technique as a means to gain access to the anterior superomedial orbit.[7] Kersten *et al*. reported a series of cases in which the vertical lid split incision was used to approach the medial intraconal space.[8] The vertical lid split approach provides a wide surgical field without the need for excessive globe traction or extensive tissue dissection. The incision heals well with no lid dysfunction and good cosmesis. We believe that the vertical lid split approach may be a useful addition to the surgical armamentarium for ONSD.

## References

[1] DeWecker L (1872). On incision of the optic nerve in cases of neuroretinitis. Rep Int Ophthalmol Congr.

[2] Friedman DI (2004). Pseudotumor cerebri. Neurol Clin N Am.

[3] Galbraith JEK, Sullivan JH (1973). Decompression of the perioptic meninges for relief of papilledema. Am J Ophthalmol.

[4] Kersten RC, Kulwin DR (1993). Optic nerve sheath fenestration through a lateral canthotomy incision. Arch Ophthalmol.

[5] Tse DT, Nerad JA, Anderson RL, Corbett JJ (1988). Optic nerve sheath fenestration in pseudotumor cerebri: A lateral orbitotomy approach. Arch Ophthalmol.

[6] Pelton RW, Patel BCK (2001). Superomedial lid crease approach to the medial intraconal space: A new technique for access to the optic nerve and central space. Ophthal Plast Reconstr Surg.

[7] Smith B (1966). The anterior surgical approach to orbital tumors. Trans Am Acad Ophthalmol Otolayngol.

[8] Kersten RC, Kulwin DR (1999). Vertical lid split orbitotomy revisited. Ophthal Plast Reconstr Surg.

